# AscF in the mycobacterial CIII–CIV supercomplex lacks metal and nucleotide binding but links malate oxidation to respiration

**DOI:** 10.1002/1873-3468.70414

**Published:** 2026-07-21

**Authors:** Eni Rile, Riccardo Diamanti, Daniel Lundin, Soni Sharma, Robin Lissner, Coenraad P. Kuil, Martin Högbom, Dirk Bald, Dan Sjöstrand

**Affiliations:** ^1^ Molecular Microbiology Section, A‐LIFE and AIMMS, Faculty of Science Vrije Universiteit Amsterdam The Netherlands; ^2^ Department of Biochemistry and Biophysics and Science for Life Laboratory Stockholm University Sweden; ^3^ Medical Microbiology and Infection Control (MMI) Amsterdam University Medical Center Location VUmc The Netherlands

**Keywords:** malate oxidation, *Mycobacterium smegmatis*, respiratory supercomplex, TPM domain

## Abstract

In most Actinobacteria, the respiratory complexes CIII and CIV form an obligate supercomplex, but the exact subunit composition varies. Here, we have characterized AscF (MSMEG_4692), a subunit of the *Mycobacterium smegmatis* CIII‐CIV supercomplex. We showed that AscF and the small, membrane‐anchored AscG constitute a heteromeric TPM domain featuring a noncanonical topology. Biophysical analysis demonstrated that the isolated AscF/AscG module lacked intrinsic affinity for metals or respiratory nucleotides *in vitro*. Functionally, an *ascF* frameshift mutant exhibited abolished malate‐dependent oxygen consumption and severe growth defects on nonfermentable energy sources. We conclude that AscF likely is not a sensor for metal ions or nucleotides but acts as an adapter subunit facilitating electron transfer from the tricarboxylic acid cycle to the mycobacterial respiratory supercomplex.

## Graphical abstract

The mycobacterial CIII‐CIV respiratory supercomplex is an obligate assembly, encompassing several subunits of unknown functions. We have characterized the intracellular subunit AscF, and show that it is unlikely to be a sensor for metals or nucleotides, but is required for growth on nonfermentable energy sources, and likely works as an adapter for connecting the TCA cycle to the supercomplex.

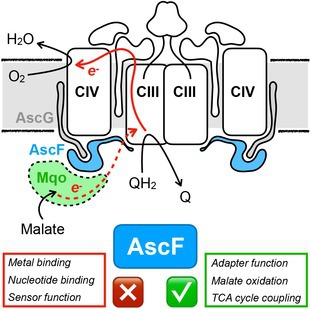

## Abbreviations

**ACMA**, 9‐amino‐6‐chloro‐2‐methoxyacridine

**ADC**, albumin/dextrose/catalase

**ADP**, adenosine diphosphate

**AMP**, adenosine monophosphate

**Asc**, Actinobacterial supercomplex

**ATc**, anhydrotetracycline

**ATP**, adenosine triphosphate

**BCA**, bicinchoninic acid

**CRISPR/Cas9**, clustered regularly interspaced short palindromic repeats/CRISPR‐associated protein 9

**cryo‐EM**, cryo‐electron microscopy

**DSF**, differential scanning fluorimetry

**GTDB**, Genome Taxonomy Database

**GTP**, guanosine triphosphate

**HEPES**, 4‐(2‐hydroxyethyl)‐1‐piperazineethanesulfonic acid

**His‐tag**, polyhistidine tag

**HRP**, horseradish peroxidase

**KEGG**, Kyoto Encyclopedia of Genes and Genomes

**MES**, 2‐(N‐morpholino)ethanesulfonic acid

**MOPS**, (3‐(N‐morpholino) propanesulfonic acid)

**Mqo**, malate:quinone oxidoreductase

**NAD+**, oxidized nicotinamide adenine dinucleotide

**NADH**, reduced nicotinamide adenine dinucleotide

**Ni‐NTA**, nickel‐nitrilotriacetic acid

**NTMH**, N‐terminal transmembrane helix

**PBS**, phosphate‐buffered saline

**PBS‐T**, phosphate‐buffered saline containing Tween‐20

**PCR**, polymerase chain reaction

**PVDF**, polyvinylidene fluoride

**qPCR**, quantitative polymerase chain reaction

**SC**, supercomplex

**SDS/PAGE**, sodium dodecyl sulfate/polyacrylamide gel electrophoresis

**SEC**, size exclusion chromatography

**sgRNA**, single‐guide ribonucleic acid

**TCA**, etricarboxylic acid

**TMB**, 3,3′,5,5′‐tetramethylbenzidine

**TPM**, TLP18.3, Psb32, and MOLO‐1

**TXRF**, total‐reflection X‐ray fluorescence

Living organisms catabolize organic compounds to extract energy, and the final steps in the aerobic version of this process are normally performed by the membrane protein complexes of the respiratory chain. Typically, membrane protein dehydrogenases (e.g., complexes I‐II) reduce quinone molecules that in turn transfer electrons to a quinol oxidase (complex III). A cytochrome *c* protein then shuttles the electrons to cytochrome *c* oxidase (complex IV), which reduces O_2_ into H_2_O. This electron transfer drives the pumping of protons, and the electrochemical gradient created is used for production of ATP by ATP synthase (complex V).

The catalytic mechanism and molecular structure of respiratory chain complexes have been extensively studied, and it has been shown that the complexes can form larger assemblies: Supercomplexes and respirasomes [1, 2] and a series of structures have provided details of their composition and organization [3, 4, 5, 6, 7, 8]. However, the functional advantages of a supercomplex, and the factors contributing to its stability and functionality still remain largely unclear.

In the phylum *Actinomycetota* (commonly called Actinobacteria), the respiratory complexes III and IV are permanently assembled in an obligate supercomplex. The Actinobacteria encompass species important for industrial processes (e.g., *Streptomyces sp*., *Corynebacterium glutamicum*) as well as human pathogens (e.g., *Mycobacterium tuberculosis, Mycobacterium leprae*), and this obligate supercomplex is thus of interest for both fundamental research and applications in biomedicine or biotechnology. The respiratory chain complexes of mycobacteria are important drug targets, e.g. for tuberculosis and Hansen's disease [9]; in particular, the complex III‐IV supercomplex has been validated as target of the imidazopyridine class of drugs [10]. Infectious species of mycobacteria are difficult and dangerous to work with, and therefore, the nonpathogenic model bacterium *Mycobacterium smegmatis* is often used for biochemical studies and protein expression [11, 12]. A deletion strain of *M. smegmatis* complex III (∆*qcrCAB*) can be functionally complemented by *M. tuberculosis* complex III, suggesting that the *M. smegmatis* supercomplex is an adequate model system for drug development against *M. tuberculosis* [13].

**Fig. 1:**
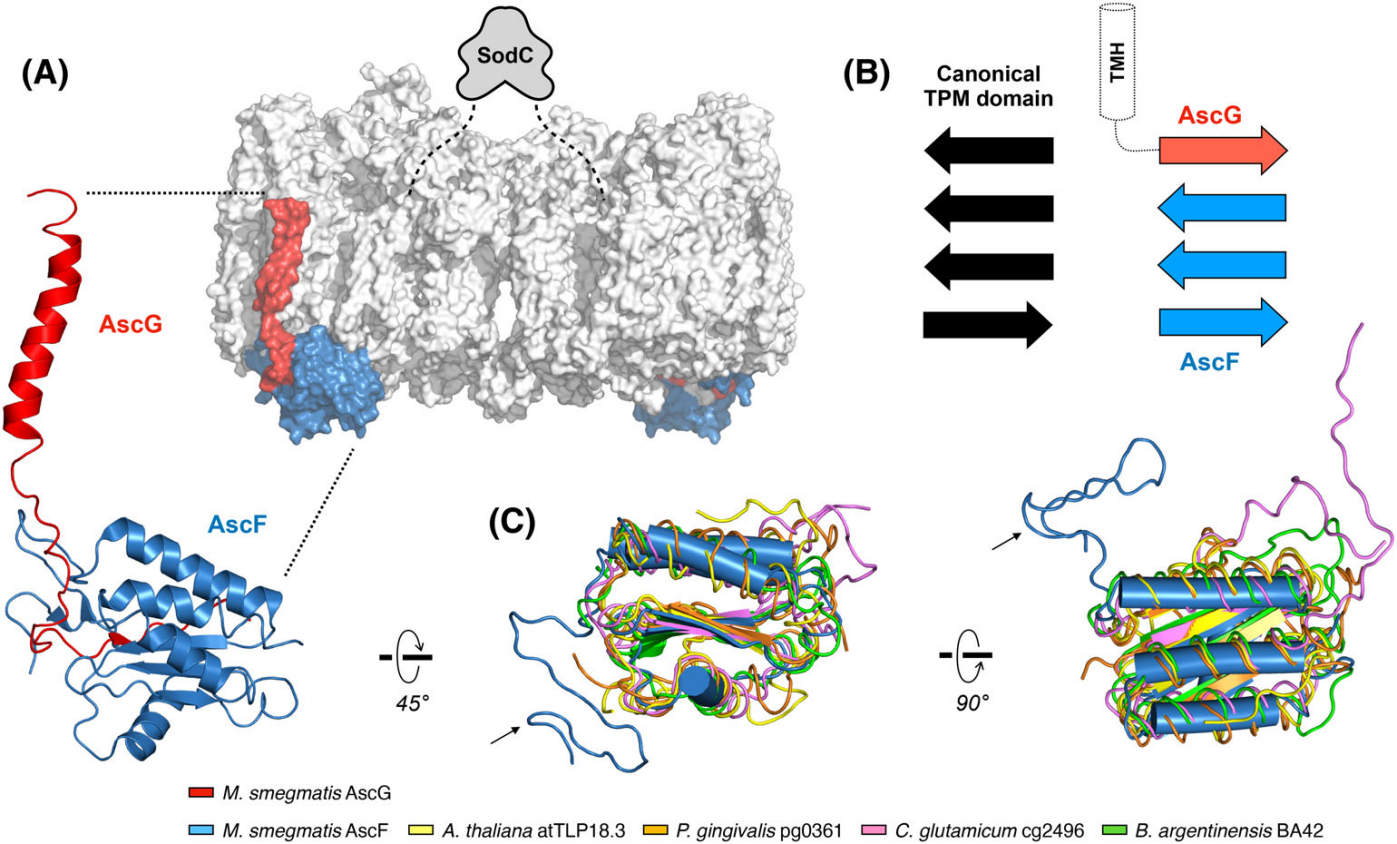
Structure and fold conservation. (A) The position of *M. smegmatis* AscG (red) and AscF (blue) in the respiratory supercomplex. (B) Beta‐sheet topology comparison. The AscF beta‐sheet is completed by the single, antiparallel beta‐strand of AscG. (C) Structural alignment (Software: DALI server) of AscF with four other experimentally determined TPM (TLP18.3, Psb32, and MOLO‐1) domain structures. The core TPM domain fold is structurally well conserved.

Several recent structures of the obligate respiratory supercomplex from *M. smegmatis* (and one from *M. tuberculosis*) reveal a number of novel idiosyncratic features [8, 14, 15, 16]. These include a di‐heme cytochrome *cc* with a transmembrane helix anchor that forms an integral part of the complex, structural adaptations in major subunits, and accessory subunits that are not part of the canonical complex III or complex IV [8, 15]. In addition to a superoxide dismutase (MSMEG_0835) on the periplasmic side of the supercomplex and the LpqE subunit (MSMEG_6078), located at the interface of the complex III and complex IV parts of the supercomplex, these accessory subunits also include a largely hydrophilic subunit located at the cytoplasmic side of the supercomplex (Fig. 1A). This subunit, MSMEG_4692, has been referred to as either CtaI [15] or AscF [17]. In our current report, we use the ‘Asc’ terminology (Asc = **A**ctinobacterial **s**uper**c**omplex [17]) to denote these accessory subunits. AscF encompasses a TPM (TLP18.3, Psb32, and MOLO‐1) domain fold and is intertwined with an extended loop of another accessory subunit located on the cytoplasmic side, AscG (MSMEG_4693, CtaJ), which inserts into the cytoplasmic membrane with a transmembrane helix.

By the time we commenced our work, the functional implications of these novel subunits were unknown. We set out to investigate the structural and biochemical properties of AscF; to find out whether it could bind metals or nucleotides (e.g., for catalytic/regulation); and to assess its biological function in the cell. Recently published structural work revealed that the membrane‐associated enzyme malate:quinone oxidoreductase (Mqo) binds directly to the cytosolic side of the complex III‐IV supercomplex [18], between complex III and complex IV, and appears to be in close contact with the AscF subunit [18]. Mqo provides a direct route for transferring electrons from malate into the quinone pool [19, 20] and electron transfer from malate leads to reduction of *c*‐type hemes and molecular oxygen; but this activity was not present in absence of the complex III‐IV supercomplex [18].

In this paper, we provide a detailed description of the structure and function of the AscF subunit, with focus on the evolutionary conservation of structural features, as well as its biochemical function and role in mycobacterial respiration and cellular growth.

## Materials and methods

### Structure conservation analysis

Structure coordinates for the *M. smegmatis* supercomplex (6HWH) and 7 other TPM domain proteins; *P*. *gingivalis* pg0361 (2KW7), *A. thaliana* AtTLP18.3 (3PTJ), *B. argentinensis* BA42 (4OA3), *C. glutamicum* cg2496 (2KPT), *B. argentinensis* BA41 (5ANP), *R. marinus* Rhom172_1776 (7TBR), and *M. tuberculosis bcc:aa3* supercomplex (8HCR) were downloaded from the Protein Data Bank. The AscF polypeptide of the *M. smegmatis* supercomplex was used for structural alignment with the other structures using the DALI server [21]. Figures were rendered with PyMol (The PyMOL Molecular Graphics System, Version 3.1 Schrödinger, LLC.).

### *M. smegmatis* AscF/AscG protein production and purification

AscF/AscG was designed as a polycistronic construct (Fig. S1A,B) and synthesized codon‐optimized for expression in *E. coli*. Truncated versions were subcloned by PCR and XhoI/BamHI insertion into the C‐terminal His‐tag vector pHis d (a pET‐28 derivative) [22]. An NdeI/XhoI flanked N‐terminal His‐tag was simultaneously added by oligo annealing/ligation (Fig. S1C). T1‐resistant *E. coli* BL21 (DE3) (New England Biolabs) was used for protein expression.

Cells were cultured in terrific broth medium (Formedium) supplemented with kanamycin (25 μg/mL) in a benchtop LEX bioreactor system (Harbinger), initially at 37 °C. Expression was induced with 0.5 mm isopropyl β‐D‐1‐thiogalactopyranoside for 10 h at room temperature. Cells were harvested by centrifugation and stored at −20 °C. Cells were resuspended in buffer A (25 mm Tris–HCl pH 7.5, 200 mm NaCl) + cOmplete™ Protease Inhibitor Cocktail (Bayer) and disrupted by sonication. The lysate was cleared by centrifugation, applied to a nickel–nitrilotriacetic acid (Ni‐NTA) agarose gravity flow column (Protino), washed (buffer A + 25 mm imidazole), and eluted (buffer A + 200 mm imidazole). After concentration with Vivaspin 4 Turbo, 3000 kD cutoff (Sartorius) the sample was applied to a HiLoad 16/60 Superdex 200 prep grade size exclusion column (GE Healthcare) pre‐equilibrated with buffer A. Fractions corresponding to the pure protein were pooled together, analyzed by SDS/PAGE and western blot, aliquoted, flash‐frozen in liquid nitrogen, and stored at −80 °C. The protein concentration was measured with a Nanodrop instrument (Thermo Scientific), using the theoretical molecular weight (24 290,18 Da) and extinction coefficient at 280 nm (25 440 M^−1^ cm^−1^) for the protein complex (ProtParam) [23].

The purified complex was run on two identical NuPAGE 4–12%, Bis‐Tris SDS/PAGE gel (Invitrogen) (55 mA/gel, 50 min, NuPAGE MES SDS Running Buffer). One gel was stained with Comassie Blue, and the other transferred onto a PVDF blotting membrane (25 V, 90 min). The membrane was rinsed with deionized water, blocked (1 h, 2,5% m/v bovine serum albumin, PBS‐T buffer), washed 3 × 10 minutes (PBS‐T), and incubated 1 h with 1:10000 Pierce HisProbe‐HRP Conjugate (Thermo Scientific). The membrane was washed 3 × 10 min (PBS‐T) to remove unbound HisProbe‐HRP and developed with Pierce TMB HRP substrate (Thermo Scientific).

### Total‐reflection X‐ray fluorescence (TXRF) metal quantification

The purified complex was incubated with 2 molar equivalents of each metal ion (Mg^2+^, Ca^2+^, Mn^2+^, Fe^2+^, Co^2+^, Cu^2+,^ and Zn^2+^) for 30 min. Unbound metal ions were removed using a HiTrap desalting 5 mL column (Cytiva) according to the manufacturer's instructions. Three independent samples were quantified by TXRF using a Bruker PicoFox S2 instrument, using Ga^2+^ as internal standard.

The results were analyzed with the instrument software. Protein concentration (29.2 μm) and Ga^2+^ concentration (28.7 μm) were measured spectrophotometrically.

### Differential scanning fluorimetry

Binding of nucleotides to AscF/AscG was studied by differential scanning fluorimetry. Each sample was prepared by mixing 15 μm AscF/G complex, 1 mm nucleotide (ATP, ADP, AMP, GTP, NADH or NAD^+^), 10x SYPRO Orange Protein Gel Stain (Invitrogen), and either 1 mm Mg^2+^, 1 mm Mn^2+,^or no metal ions. The samples were measured between 22 °C and 99 °C on a StepOne™ Real‐Time PCR System (Applied Biosystems) and analyzed using Protein Thermal Shift™ Software (Applied Biosystems). Each measurement was performed in quadruplicate.

### Construction of an *ascF* frameshift mutant

The target for CRISPR/Cas9 was predicted using CHOPCHOP [24]. The sequence of the single guide RNA (sgRNA) for targeting *ascF (msmeg_4692)* is as follows: CACCATGGACCTCGTGGTGCTC. CRISPR/Cas9 frameshift mutant was constructed following the description by [25]. Electro‐competent *M. smegmatis* were electroporated at a single pulse of 2.5 kV, 25 μF, and 720 Ω using 1 μg of pCRISPRx‐Sth1‐Cas9‐L5 (Addgene 140 993) plasmid harboring the sgRNA. The cells were plated on 7H10 plates containing Kanamycin and 100 ng/mL ATc (IBA Lifesciences) after 4‐h recovery in 7H9 medium. Single colonies were picked up after 3 days for PCR amplification of the targeted locus using Phusion High‐Fidelity DNA polymerase (Thermo Fisher) with primers: CAAGTGGCAAGTGGTGACAT and ATGTACACGGCGAAACGG. Gene sequences were verified using Sanger Sequencing (Macrogen). For the replacement of pCRISPRx‐Sth1‐Cas9‐L5, mutant was electroporated with pTdTomato‐L5 (Addgene 140 994) and plated on 7H9 plates containing streptomycin.

### Construction of genetic complementation strain

Amplification of the *ascF* gene was performed using genomic DNA from *M. smegmatis* as template, Phusion High‐Fidelity DNA polymerase (Thermo Fisher) and primers: GAGGAATCACGCTAGGTGGCAAGTGGTGACATCGCG and CATACGGATAGGATCTCAGGCAGGCGAAACGC. The amplified gene was cloned into a digested pSMT3 vector using In‐Fusion cloning (Takara Bio). The construction was verified using Sanger Sequencing (Macrogen) and electroporated into the *ascF* frameshift mutant.

### Bacterial growth assay

*M. smegmatis* strains (WT, Δ*qcrCAB* mutant, the *ascF* frameshift mutant, and the *ascF* frameshift mutant complemented with the *ascF* gene) were grown at 37 °C in Middlebrook 7H9 broth containing 0.2% glycerol, 0.05% (w/v) Tween 80, and supplemented with 10% ADC (Difco). Antibiotics were added: 50 μg/mL hygromycin (Roche), 50 μg/mL kanamycin (Sigma), or 30 μg/mL streptomycin (Sigma). For measuring cellular growth, the culture was cultivated in either this 7H9 broth or in modified HdB medium [26] containing 20 mm malate, succinate, or acetate.

For measuring growth curves, cultures of the different *M. smegmatis* strains were diluted to OD_600_ 0.01 in the designated medium in 96‐well flat‐bottom microplates (Greiner Bio). The plates were incubated at 37 °C in a microplate reader (BMG Labtech) for 60 h and measured every 10 min.

### Preparation of membrane fractions

Membrane fractions from *M. smegmatis* were prepared as described previously [27]. Briefly, bacteria were harvested by centrifugation at 6000 g for 20 min and washed with PBS (phosphate‐buffered saline, pH 7.4). Each 5 g wet weight cell pellet was resuspended in 10 mL of ice‐cold lysis buffer (50 mM MOPS (3‐(N‐morpholino) propanesulfonic acid), 2 mM MgCl_2_ at pH 7.5) including protease inhibitors (cOmplete, EDTA free; protease inhibitor cocktail tablets from Roche). Lysozyme (1.2 mg ml^−1^, final conc.), 1500 U (final conc.) of deoxyribonuclease I (Invitrogen), and 15 mM MgCl_2_ (final conc.) were added. The cells were then broken by three passages through One Shot Cell Disruptor (Constant Systems) at 0.83 kb. Unbroken cells were removed by three centrifugation steps at 6000 g for 20 min at 4 °C. The membrane fractions were pelleted by ultracentrifugation at 222000 g for 1 h at 4 °C. The pellet was resuspended in lysis buffer and the protein concentration was determined using the BCA Protein Assay kit (Pierce) as described by the manufacturer.

### Oxygen respiration assays and proton pump experiments

Oxygen respiration of isolated membrane fractions was measured with a Clark‐type oxygraph (Hansatech) as described previously [28]. Briefly, the electrode was fully aerated at 37 °C and calibrated with sodium dithionite (Na_2_S_2_O_4_). NADH or malate was added as substrate to a final concentration of 500 μm and oxygen respiration was measured for 3 min. Activity values were calculated for the interval from 1.5 min to 2.5 min after addition of substrate.

Proton pump activity was measured by decrease of 9‐amino‐6‐chloro‐2‐methoxyacridine (ACMA) fluorescence, as described earlier [19]. Isolated membrane fractions (0.36 mg ml^−1^) were preincubated at 37 °C in 10 mm HEPES‐KOH (pH 7.5), 100 mm KCl, 5 mm MgCl_2_ containing 2 μm ACMA, and a baseline was monitored for 5 min with a Cary Eclipse Fluorescence spectrophotometer (Varian Inc, Palo Alto, USA). The reaction was then started by adding 5 mm succinate. After 5 min, any proton gradient was collapsed by the addition of 1 μm SF6847. Excitation and emission wavelengths were 410 and 480 nm, respectively.

### RNA isolation, reverse transcription, and qPCR analysis

RNA isolation was performed using the NucleoSpin RNA Kit (Machery Nagel (Düren, Germany)). Briefly, *M. smegmatis* was harvested at a density of 10^9^ cells/mL. RNA was isolated according to the manufacturer's instructions, with the following modifications [29]. The cells were bead‐beaten in tubes containing 0.1 mm Zirconium–silica glass beads, 500 μL Buffer RA1, and 5 μL β‐mercaptoethanol for 1 min at a speed of 6 m/s for cell lysis. The RNA concentration was determined using a NanoDrop 2000 spectrophotometer (Thermo Fisher). Reverse transcription was done with High‐Capacity cDNA Reverse Transcription Kit with RNase inhibitor (Applied Biosystems® (Waltham, MA, USA)) according to the manufacturer's protocol. The obtained cDNA was subjected to qPCR analysis using the gene‐specific primers ATGGAGTTCATCGACGACAAG and GTGCTGCGACCAGTTCA for *mqo*, ATCTCCTTGGCGAGCTCTTC and GGGCTACAAGTTCTCGACCT for *sigA* as the reference gene for normalization. The qPCR reaction was performed with the SensiFAST™ SYBR® Hi‐ROX Kit (Meridian Bioscience) as follows: 95 °C (2 min) followed by 40 cycles of 95 °C (20 s), 60.0 °C (10 s), and 72 °C (20 s). For quantification of transcriptional changes, we used the comparative 2^‐ΔΔCt^ method. The expression of *mqo* was normalized with the *sigA* transcript level.

### Analysis of the Mqo‐AscF/AscG binding interface

We used the AlphaFold 3 server [30] to model the Mqo‐AscF/AscG complex. In order to increase the focus on predicting the Mqo‐AscF/AscG interaction, we excluded parts of AscF and AscG known to interact with other parts of the supercomplex, and the N‐ and C‐terminal ends not resolved in the Cryo‐EM structure of the complex. The following sequences were used as input: Mqo T12‐V496; AscF P42‐A157; AscG P33‐W79.

### Taxonomic distribution of proteins

Sequences for AscF and AscG (previously MSMEG_4692 and MSMEG_4693, respectively) were downloaded from KEGG, and the jackhmmer tool at https://www.ebi.ac.uk/Tools/hmmer was used to create HMMER [31] profiles for the two proteins. For the longer AscF (157 aa), we used a bitscore cutoff of 100, and for the shorter AscG (79 aa), we used a cutoff of 50, for inclusion of sequences in the profiles. The AscF profile converged at 190 proteins and AscG converged at 141 proteins. The two profiles were used to search all species representative genomes from GTDB (R10‐RS226; [32]), a total of 143 614 bacterial and archaeal genomes. Genes coding for Mqo were identified with Prokka (v.1.14.6; [33]). R (v.4.5.2; [34]), Tidyverse (v.2.0.0; [35]), and Anvi'o (v.9; [36]) were used to visualize the results.

## Results

### Analysis of the AscF/G TPM domain

AscF is a cytoplasmic, membrane‐attached protein with a TPM (TLP18.3, Psb32, and MOLO‐1) domain fold and a long C‐terminal loop extending toward QcrB and an internal proton pathway (the D‐channel) of the supercomplex [8, 15]. AscG is a short protein consisting of a transmembrane alpha‐helix, a loop region, and a short beta‐strand, which completes the canonical four‐strand sheet of the TPM domain by integrating with the three strands of AscF (Fig. 1A). Notably, though, the direction of the AscG strand is opposite to the corresponding strand in the canonical topology (Fig. 1B).

The TPM domain is a structurally conserved protein fold, found across all kingdoms of life [37, 38]. However, the amino acid sequences of TPM domains are highly divergent, and many species have multiple TPM domain‐encompassing proteins. Sequence alignment of AscF and AscG with the previously studied TPM domains from distantly related species thus proved challenging (due to the very low level of primary sequence conservation).

A comparison between the structures of *Caenorhabditis elegans* MOLO‐1 and *Porphyromonas gingivalis* pg0361 identified conserved hydrophobic residues likely to be structurally important in the TPM domain core [39], and similarly conserved sets of hydrophobic amino acids are found at the cores of AscF and *Bizionia argentinensis* BA41 (Fig. S2A,B).

In stark contrast to their ubiquity, relatively few TPM domain‐containing proteins have been properly characterized, and only seven other structures have been determined to date (the near‐identical *M. tuberculosis* AscF included). Despite sharing a conserved core ‘α‐β‐α “sandwich” fold’ (Fig. 1C), the proteins differ in the arrangement of secondary structure elements, mainly in the N and C termini (Fig. S3A–F). Interestingly, *B. argentinensis* BA42 features a truncated N terminus where the missing beta‐strand is structurally compensated by its own C‐terminal tail (Fig. S3D). This alternative arrangement is analogous to the recruitment of the AscG strand in the AscF/AscG complex, highlighting a significant evolutionary flexibility in how the TPM domain beta‐sheet can be completed. The long N‐terminal extension of AscF is absent in the other structures (Fig. 1C, Fig. S3A‐F), except for the nearly identical *M. tuberculosis* protein (Fig. S3G).

TPM domains are present in a range of functionally diverse proteins. For example, *M. tuberculosis* Rv2345 was proposed to be an acid phosphatase [40] while *Caenorhabditis elegans* MOLO‐1 likely functions as a positive regulator of acetylcholine receptors in the neuromuscular junction [39]. The structure of *Arabidopsis thaliana* TLP18.3 [41] reveals a single Ca^2+^ ion bound close to the (cocrystallized) 0‐phospho‐L‐serine binding pocket, likely implied in its phosphatase activity. The structure of *Rhodobacter marinus* Rhom172_1776 (a protein of unknown function) encompasses an Mg^2+^ ion [42], The otherwise structurally very similar *B. argentinensis* BA41 was shown to have ATPase activity, but does not bind any metals [43]. However, the stand‐alone TPM domain protein BA42 (function unknown) from the same species binds two Ca^2+^ ions, suggested to be involved in activity regulation [44]. The Ca^2+^‐binding residues of *Arabidopsis thaliana* TLP18.3 and *B. argentinensis* BA41 are located on opposite sides of the TPM domains and are not conserved in the AscF/AscG structure (Fig. S2B).

Binding of metal ions and/or nucleotides to AscF/AscG could potentially have a role in regulation of the supercomplex, as these subunits are located close to a proton entry channel. Interestingly, binding of ATP and feedback inhibition of respiratory activity has been reported for mitochondrial complex IV [45]. Although ATP typically binds to domains harboring a characteristic alpha/beta twist fold [46], binding of ATP to proteins lacking this fold has been reported earlier, for example for subunit epsilon of bacterial ATP synthase, which upon ATP binding acts as a sensor for regulating ATP hydrolysis and synthesis activity [47].

### Evaluation of metal and nucleotide binding to the AscF/AscG complex

**Fig. 2:**
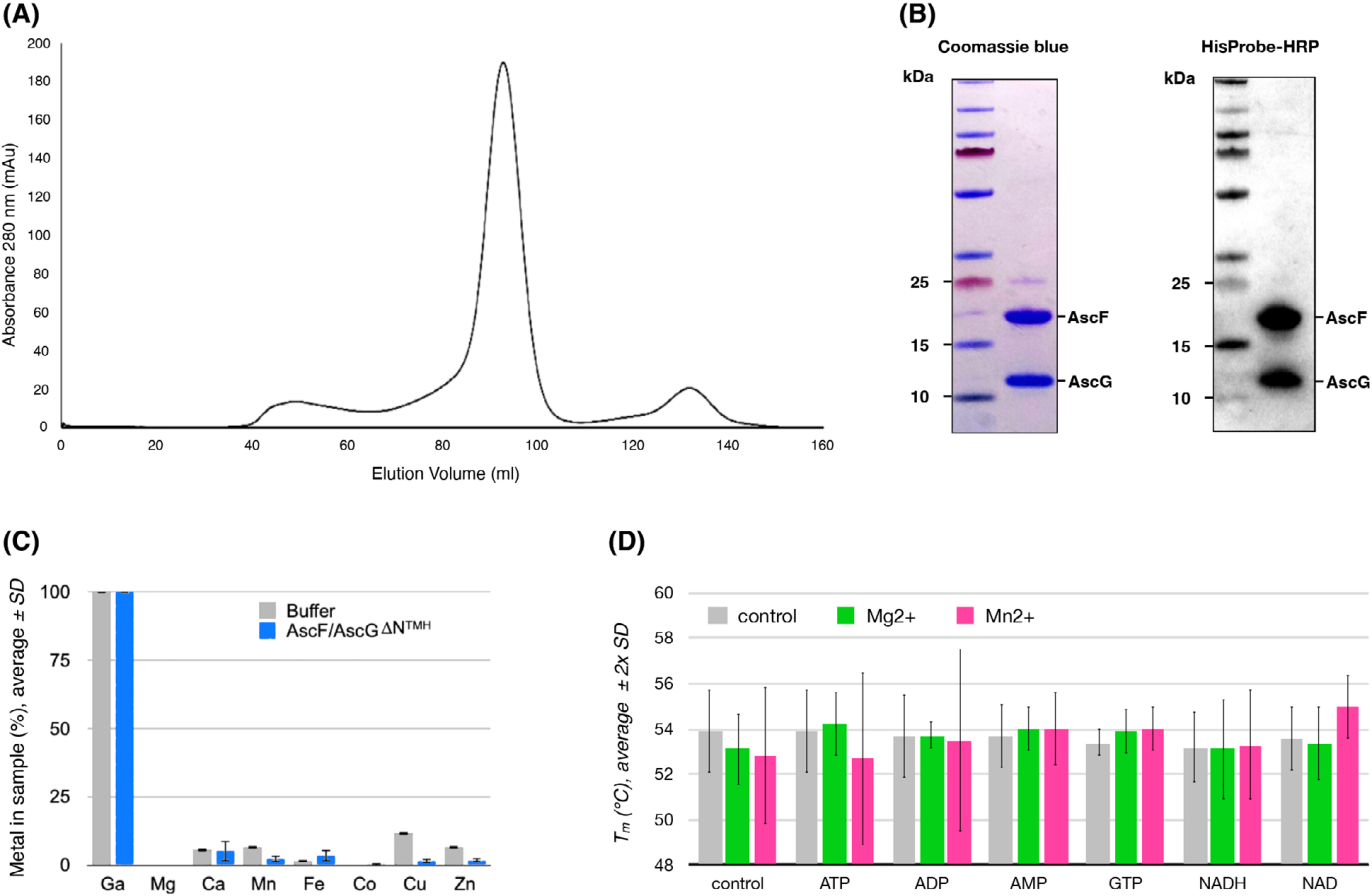
Purification and characterization of the helix‐less AscF/AscG complex. (A) Size exclusion chromatogram for the purified *M. smegmatis* helix‐less (∆NTMH‐) AscF/AscG complex. (B) SDS/PAGE of the ∆NTMH‐AscF/AscG complex. The Coomassie stained gel (left panel) shows 3 visible bands around 12 kDa, 18 kDa, and 25 kDa. The dominant bands around 12 kDa and 18 kDa correspond to AscF and AscG, respectively. The weak ~25 kDa band is not visible in the western blot (anti‐His, right panel) and is likely a contaminant. The main peak in the chromatogram was used for the total‐reflection X‐ray fluorescence (TXRF) and differential scanning fluorimetry (DSF) assays. However, since the 25 kDa contaminant is potentially the reason for the shoulder around 83 mL elution volume, the first third of the peak was excluded when SEC (size exclusion chromatography) fractions were pooled for further experiments. (C) TXRF analysis of metal binding to the ∆NTMH‐AscF/AscG complex. Metal binding is shown as the fraction of protein that binds the respective metal. Data are presented as mean ± SD from three independent experiments. Gallium (1:1 metal:protein binding) was used as an internal standard. (D) Binding of 6 nucleotides involved in cellular respiration and/or signaling to ∆NTMH‐AscF/AscG; assayed by DSF. Data are presented as mean ± 2x SD from four experiments.

To evaluate whether the AscF/AscG domain can bind metal ions or nucleotides, we recombinantly expressed the *M. smegmatis* AscF/AscG complex in *E. coli* (with the transmembrane helix of AscG removed). Purification of the resulting ∆NTMH‐AscF/AscG complex by Ni‐NTA His‐tag affinity chromatography followed by size‐exclusion chromatography yielded pure protein, as assessed by Coomassie staining and anti‐His‐tag western blot (Fig. 2A,B). The purified complex eluted as one major peak from the size exclusion column, indicating that these two subunits can form a stable AscF/AscG complex.

We examined the metal binding properties of the AscF/AscG complex using total‐reflection x‐ray fluorescence (TXRF). The protein was soaked with 2:1 (metal:protein) molar equivalents of Mg^2+^, Ca^2+^, Mn^2+^, Fe^2+^, Co^2+^, Cu^2+,^ and Zn^2+^ (this subset of metal ions was selected for being the most biologically relevant cations). None of the metals tested bind at a concentration higher than 10% of the protein concentration (Fig. 2C). Small amounts of residual metal are likely due to metal carryover from the desalting step.

We also investigated the possibility of nucleotide binding to AscF/AscG. The binding of nucleotides was assayed by differential scanning fluorimetry (DSF). Six nucleotides, all involved in cellular respiration and/or signaling, were included in the study. The AscF/AscG complex was incubated with one of the six nucleotides (ATP, ADP, AMP, GTP, NADH, and NAD^+^). Binding was also tested in the presence of manganese and magnesium ions (which are known to be needed for binding of nucleotides). The average Tm for each condition (Fig. 2D) was calculated to be between 52,6 ± 3.8 °C and 55 ± 1.4 °C. The differences in Tm were not statistically significant between the tested samples, showing that, at least in an isolated *in vitro* context, AscF/AscG does not bind these nucleotides.

### Importance of AscF for malate oxidation activity

The observed binding of Mqo to the complex III‐complex IV supercomplex via AscF [18] led us to hypothesize that the AscF subunit has a functional role in malate utilization. To address this question, we inactivated the *ascF* gene in *M. smegmatis*. We used the *Streptococcus thermophilus* CRISPR1‐Cas9 (Sth1Cas9) gene editing tool, which allows for introduction of frameshift mutations upon repair of double strand breaks by nonhomologous end joining [25]. We transformed *M. smegmatis* with the pCRISPRx‐Sth1Cas9‐L5 plasmid expressing a suitable single guide RNA targeted to the 5′ end of *ascF*. DNA sequencing of the targeted locus in the resulting colonies confirmed the presence of small insertions and deletions (indels) at the expected cleavage site. We selected a clone harboring an out‐of‐frame mutation (‘ascF‐fs mutant’) for further characterization (Fig. S4).

**Fig. 3:**
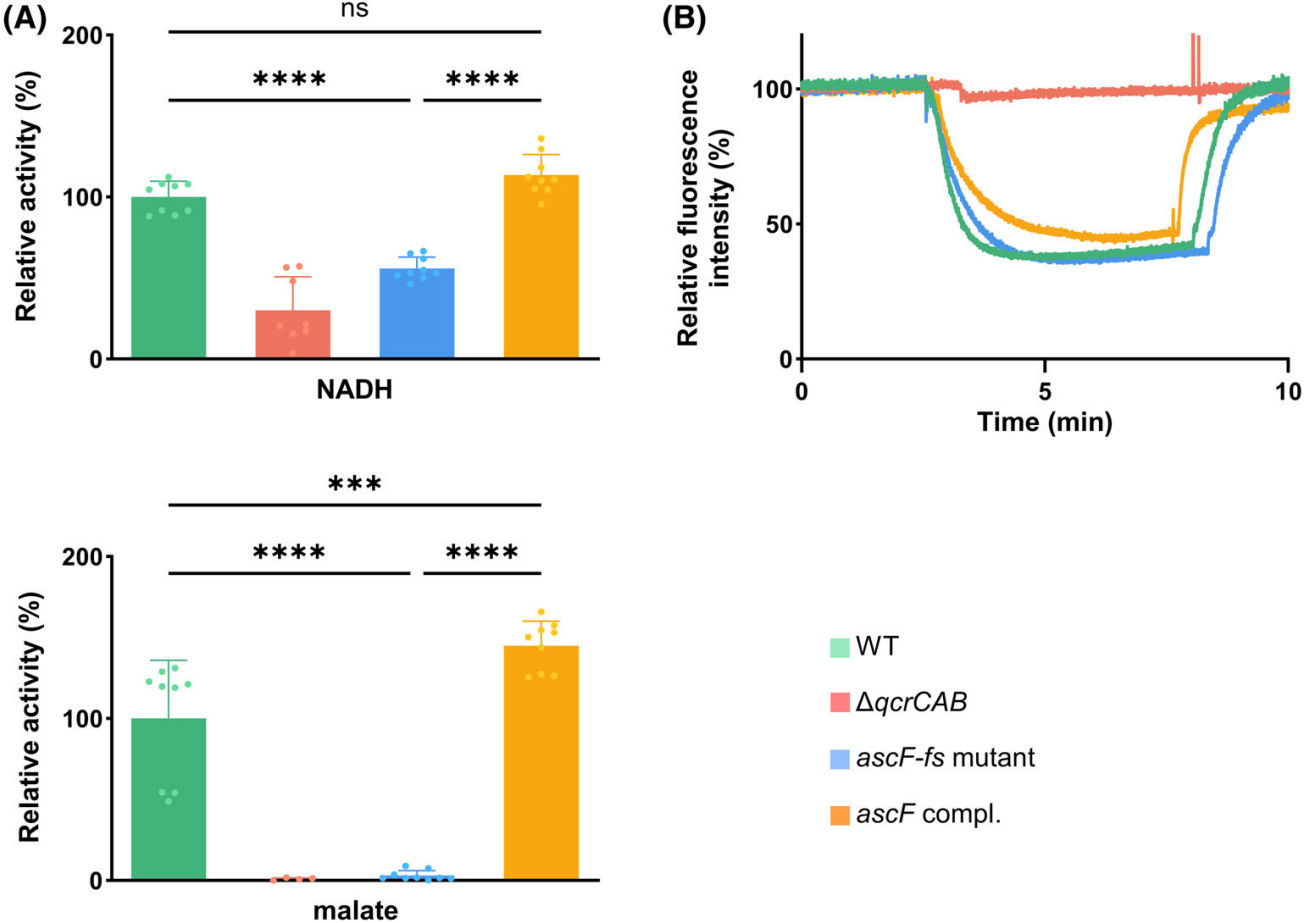
Oxygen consumption and proton pumping activity of membrane fractions. (A) Oxygen consumption activity monitored by Clark‐type electrode. Oxygen consumption rates of membrane vesicles prepared from the indicated strains were measured using either NADH (top) or malate (bottom) as electron donors. Activity of the wild‐type strain (green) was normalized to 100%. Data are presented as mean ± SD from three independent experiments. Statistical significance was determined by one‐way ANOVA followed by Tukey's multiple comparisons test. ns, not significant, *P* ≥ 0.05, ***, *P* < 0.001, ****, *P* < 0.0001. (B) ACMA (9‐amino‐6‐chloro‐2‐methoxyacridine)‐based fluorescence assay monitoring proton pumping activity. Isolated membrane fractions from the indicated strains were energized with succinate to initiate proton pumping, as monitored by quenching of the fluorescent dye ACMA. Fluorescence recovery was induced by addition of the protonophore SF6847. Data are representative traces from three independent experiments.

Subsequently, we assessed whether the AscF subunit is needed for oxidation of malate, as suggested by the structural association of Mqo with the supercomplex through AscF. We measured oxygen consumption activity of membrane vesicles isolated from *M. smegmatis* using either NADH or malate as electron donor. Oxygen consumption rates of the wild‐type strain were 81 nmol O_2_ * min^−1^ * mg^−1^ and 49.6 nmol O_2_ * min^−1^ * mg^−1^ with NADH and malate as substrate, respectively, which are in the same range as previously reported values [18, 28, 48, 49]. While NADH‐driven respiration in the *ascF‐fs* mutant was partially preserved (approximately 60% of wild‐type level), malate‐dependent oxygen consumption activity was not detectable (Fig. 3A). The genetically complemented strain restored malate‐driven activity. We confirmed that the *mqo* gene is still expressed in the *ascF‐fs* mutant, although the expression levels were slightly lower as compared to wild‐type (Fig. S5). To assess whether the observed lack of malate‐driven oxygen consumption activity is caused by impaired intactness of the supercomplex in the *ascF*‐fs mutant, we measured the proton pumping activity. For this purpose, we used the pH‐sensitive dye ACMA, whose fluorescence is quenched upon acidification of membrane vesicles due to proton pumping. The *ascF‐fs* mutant displayed comparable proton pumping activity to the wild‐type strain in terms of both maximum degree of fluorescence quenching and rate of quenching (Fig. 3B, Fig. S6). As a control, for a mutant with deleted *qcrCAB* genes [50], which encode the core subunits of complex III, no activity was detectable in this assay. Taken together, these results reveal that the AscF subunit indeed is required for malate‐driven respiratory activity.

### Importance of AscF for growth

**Fig. 4:**
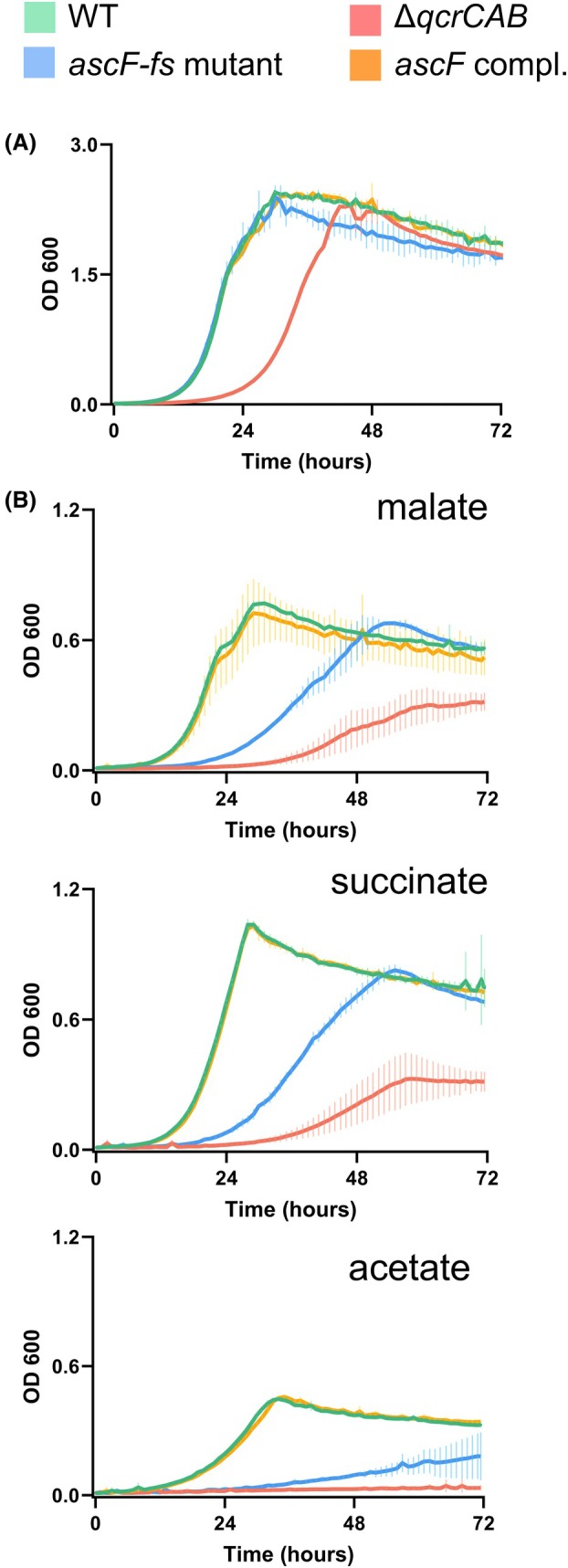
Growth of *M. smegmatis* with disrupted *ascF* gene. Growth of *M. smegmatis* wild‐type (green), Δ*qcrCAB* mutant (red), *ascF‐fs* mutant (blue), and the *ascF‐fs* mutant complemented strain (orange) cultured in (A) 7H9 medium supplemented with ADC (albumin/dextrose/catalase) or (B) in HdB medium supplemented with malate, succinate, or acetate as the sole energy source. Data are presented as mean ± SD from three independent cultures.

In *M. smegmatis* a Δ*mqo* mutant displayed delayed growth on fermentable energy sources and was unable to grow on nonfermentable carbon sources [48]. Therefore, we assessed growth of the *ascF‐fs* mutant under various growth conditions. The *ascF‐fs* mutant showed growth comparable to the wild‐type strain when cultured in standard medium with fermentable energy sources (7H9 medium supplemented with albumin/dextrose/catalase). As a control, an *M. smegmatis* strain with deleted *qcrCAB* genes exhibited clearly attenuated growth (Fig. 4A), as shown earlier [28, 50]. However, growth of the *ascF‐fs* mutant in minimal medium (HdB) supplemented with either malate, succinate, or acetate as energy source exhibited markedly reduced growth, which was restored to wild‐type level again upon complementation with the *ascF* gene (Fig. 4B). These results reveal that the AscF subunit is important for growth in the presence of nonfermentable energy sources.

### Analysis of the Mqo‐AscF binding interface

The previously published cryo‐EM structure of Mqo and the CIII‐CIV supercomplex [18] is of low resolution (~6–9 Å for the Mqo density) and did not reveal any details of the binding interface other than the general arrangement of secondary structure components. We therefore let AlphaFold 3 [30] predict a model of the Mqo‐AscF/G complex, with the AscG TM helix and AscF C‐terminal loop removed from the respective input sequences (since we know from previous structures that those interact with other parts of the SC), as well as the N‐ and C‐terminal Mqo residues not resolved in the available structure. The resulting model is remarkably similar to the model previously derived from the low‐resolution cryo‐EM data, with a binding interface encompassing two alpha‐helices from AscF and one helix, two short loops and a beta‐strand from Mqo (Fig. S7A,B).

A closer look at the AlphaFold 3 model of the binding interface reveals a number of potential residue‐residue contacts (Fig. S7C). Two areas in the interface stand out: 6 charged residues (3 from each side) forming a ‘zipper’, and a 6‐residue hydrophobic core (also 3 from each protein). Side chain distances in the two areas are consistent with polar and hydrophobic contacts, respectively. While this analysis is based on a computer‐generated model, the previously published experimental data independently supports the same general arrangement of the binding interface. This concordance indicates that the approximate positions of the residues in the interface, including the putative charged ‘zipper’ and hydrophobic core, are likely correct. In combination, the data thus support a strong putative interface for direct interaction between Mqo and AscF.

### Taxonomic distribution of *ascF* , *ascG,* and *mqo*

**Fig. 5:**
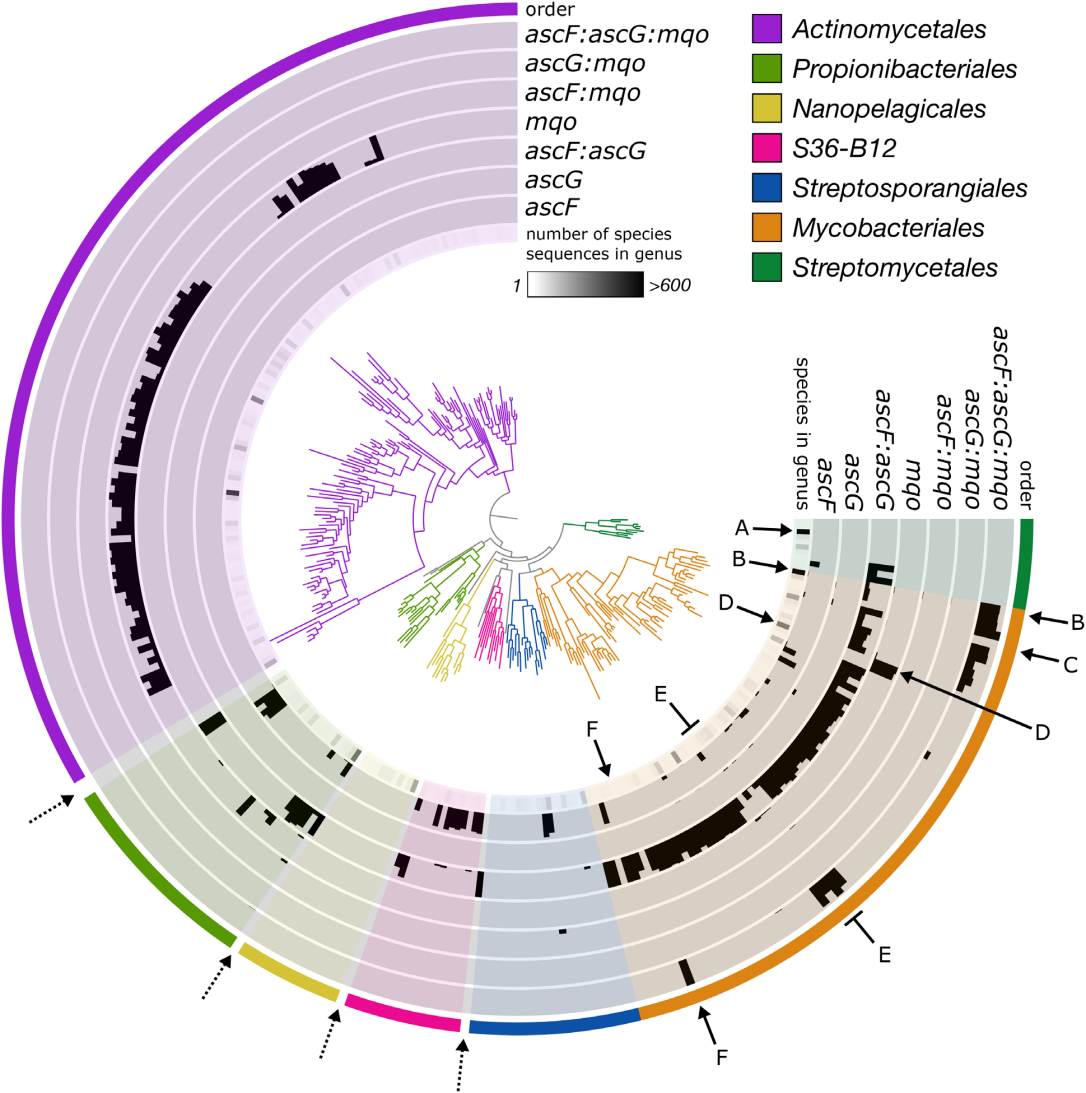
Taxonomic distribution of *ascF*, *ascG*, and *mqo* in *Actinomycetota*. Taxonomic distribution (Software for visualization: Tidyverse, Anvi'o) of *ascF*, *ascG*, and *mqo* (*ascF* and *ascG* were predominantly found in the class *Actinomycetia*, and the tree was therefore reduced to this class). The sizes of black bars represent the fraction of species in the genus that harbor at least one copy of the gene (or combination of genes). The tips of the tree branches represent genera, with colors representing the orders of *Actinomycetia* (except for four orders with very few species in a single genus; dashed arrows in the figure). Data for the species number ‘heat map’ ring was capped to 600 species for clarity: The shade of the bar for a genus indicates the number of species sequences included for the genus (darker = higher number of species sequences). None of the three genes were found in *Nanopelagicales*, and only *mqo* in *Actinomycetales*. A: None of the genes were found in *Streptomyces* spp. (> 1500 species). B: All proteins were found in most *Mycobacterium* spp. C: No *mqo* was found in *Tsukamurella* spp. D: Neither *ascF* nor *ascG* were found in *Corynebacterium* (and *Dietzia*) spp. E: All genes were found in most or all *Blastococcus, Modestobact*er, Geodermatophilus and Klenkia spp. F: All genes were found in *Proteofrankia* spp.

In total, we found 5909 *ascF* and 2877 *ascG* genes (in 5846 and 2865 genomes, respectively). Both were predominantly found in the phylum *Actinomycetota* (3547 and 2877 hits respectively for *ascF* and *ascG)*, with *ascG* being exclusive to this phylum. Both *ascF* and *ascG* are most abundant in the order *Mycobacteriales* (Fig. 5A–F), and neither was found in the orders *Actinomycetales* and *Nanopelagicales*. *ascG* was (almost) exclusively found in combination with *ascF*, while the latter was also found separately.

The *mqo gene* was found in several bacterial phyla, with the highest relative abundance in *Campylobacterota* (60% of species). In *Actinomycetota*, we found 3393 *mqo* sequences (20% of species). Contrary to *ascF* and *ascG*, *mqo* is highly abundant in the order Actinomycetales (but not Nanopelagicales). Somewhat unexpectedly, *mqo* is missing in most genera of *Mycobacteriales*, except for the *Blastococcus/Modestobacter/Geodermatophilus/Klenkia* branch (and, separately, *Proteofrankia* spp) (Fig. 5E,F).

As expected, *mqo*, *ascF*, and *ascG* were predominantly found in combination in the *Mycobacterium*‐encompassing branch of *Mycobacteriales*, with the exception of *Tsukamurella* (Fig. 5B,C).

Interestingly, *Corynebacterium* (and the neighboring genus *Dietzia*) harbor *mqo*, but are missing both *ascF* and *ascG*, unlike most other *Mycobacteriales* genera (Fig. 5D). This explains the observed differences in architecture between the *M. smegmatis* and *C. glutamicum* respiratory supercomplexes [17] and suggests that the *Corynebacterium*/*Dietzia* genera have lost these genes.

The *Streptomyces* genus has a complex III‐IV supercomplex [51], but neither *ascF*/*ascG* nor *mqo* was found in *Streptomyces* (>1500 species) (Fig. 5A). This suggests that the *Streptomyces* supercomplex has a different architecture than the previously known *Mycobacterium* and *Corynebacterium* structures. Apparently, the combination of *mqo* and the *ascF*/*ascG* module provides an evolutionary advantage for mycobacteria and many of its closely related families, while other orders of Actinomycetia have evolved other variations in functional architecture, likely due to different environmental conditions or carbon source availability.

## Discussion

The AscF/AscG complex does not bind metal ions in our experiments, which is in line with our observations that the protein does not contain the metal coordination sites found in other known TPM domains, and also shows that there is no novel, alternative metal binding site in the TPM domain of AscF/AscG. The lack of metal binding also suggests that AscF/AscG does not have phosphatase activity. As our DSF data do not support a function of AscF as a nucleotide binding protein, it is also unlikely for AscF to be a cytoplasmic sensor for ATP or other nucleotides. However, since these assays were performed with the isolated AscF/AscG globular domain, metal or nucleotide binding dependent on local conditions or interactions with the rest of the supercomplex cannot be completely excluded. As the *ascf‐fs* mutant displayed decreased oxygen consumption activity while maintaining wild‐type level of proton pumping, a role of AscF in regulating proton flux may be feasible, consistent with the location of this subunit close to a proton entry channel.

We found that lack of the AscF subunit abolishes malate‐driven oxygen consumption by the mycobacterial respiratory chain. This finding extends previous structural and functional data that revealed electron transfer from malate via *c*‐type heme groups to molecular oxygen and showed Mqo bound to the respiratory supercomplex in proximity to AscF [18], and highlights the critical role of this subunit in coupling malate oxidation to electron transport. AscF likely acts as a docking platform for Mqo and stabilizes Mqo–supercomplex interactions. As such, AscF may constitute an important functional link between the respiratory chain and the TCA cycle. How electrons from a malate molecule bound within Mqo are transferred to the respiratory supercomplex, and how this process is integrated with canonical electron flow from a menaquinol molecule onto molecular oxygen, remains to be investigated. Usage of specific accessory subunits to impart new functionalities on a respiratory complex has been reported earlier. As an example, structural and functional studies on the NADH dehydrogenase (complex I) from the cyanobacterium *Thermosynechococcus elongatus* revealed that a photosynthesis‐specific subunit, NdhS, is instrumental for the interaction with ferredoxin, thereby linking complex I to cyclic electron flow from photosystem I [52]. Investigation of idiosyncratic subunits of membrane protein complexes with currently unknown function might therefore lead to unexpected new insights.

The taxonomic distribution of *ascF*/*ascG* correlates well with *mqo* in *Mycobacterium* spp. and closely related genera, with a majority of species in most genera encompassing all three proteins. However, the distribution of *ascF*/*ascG* does not correlate with *mqo* in the other branches of *Mycobacteriales* (and the other orders of *Actinomycetia*). This indicates that AscF/AscG may have a different function in species that lack Mqo and that its role as an Mqo connector in the *Mycobacterium* [18, 48] branch of *Mycobacteriales* could be a later evolutionary adaptation. The taxonomic distribution further suggests that *ascF*/*ascG* was lost in the *Corynebacterium/Dietzia* branch, indicating that the *M. smegmatis* architecture likely represents the original version of the supercomplex in the order *Mycobacteriales*.

While the *ascF‐fs* mutant showed no growth defect in nutrient‐rich 7H9 medium, its growth was severely attenuated in HdB medium supplemented with malate, succinate, or acetate as non‐fermentable energy sources. The *ascF‐fs* mutant growth phenotype is however less pronounced than for the Δ*mqo* mutant, which showed impaired growth even with fermentable energy sources and did not display any detectable growth in the presence of non‐fermentable energy sources [48]. This difference in growth phenotype supports the view that Mqo can transfer electrons from malate to the quinone pool via more than one route; either by anchoring to the complex III‐IV supercomplex mediated by AscF, or alternatively by a route that likely includes direct binding to the cytoplasmic membrane (or weak/transient binding to the AscF‐less supercomplex) and subsequent electron transfer to the quinone pool.

The strong growth reduction of the *ascF‐fs* mutant on nonfermentable energy sources might be of particular importance for pathogenic mycobacterial strains. During *M. tuberculosis* infection, nonfermentable energy sources play a central role in bacterial persistence and virulence. *M. tuberculosis* residing in human macrophages mainly utilizes host‐derived fatty acids as a nonfermentable energy supply instead of fermentable energy sources, resulting in increased dependency on the respiratory chain and ATP synthase [53, 54, 55]. Unlike *M. smegmatis*, *M. tuberculosis* harbors not only Mqo for malate oxidation but also the NAD^+^‐dependent malate dehydrogenase Mdh [48]. Nevertheless, Mqo has been shown to be critical for *M. tuberculosis* survival during infection in macrophages and in mouse lungs [56]. A direct structural connection of Mqo to the respiratory chain, with AscF as the linker, may represent a mechanism to optimize energy extraction from lipid‐rich host environments.

In summary, our results suggest that AscF represents a noncanonical accessory subunit that is important for maintaining a molecular framework through which the respiratory chain organization influences mycobacterial fitness under metabolically restrictive conditions.

## Author contributions

E.R., R.D., and S.S. performed experiments; E.R., R.D., R.L., and D.S. designed experiments and/or analyzed data; C.P.K., M.H., D.B., and D.S. supervised and coordinated experiments; E.R., R.D., M.H., D.B., and D.S. wrote the manuscript with contributions from all co‐authors.

## Conflict of interest

The authors declare no conflict of interest.

## Supporting information


**Fig. S1.** Expression constructs for protein purification.
**Fig. S2.** TPM domain hydrophobic core and metal binding comparison.
**Fig. S3.** Structural alignments with other TPM domain proteins.
**Fig. S4.** Schematic representation of the *ascF* frameshift mutation.
**Fig. S5.** qPCR analysis of *mqo* expression.
**Fig. S6.** Quantification of proton pumping activity from ACMA fluorescence traces.
**Fig. S7.** Analysis of the binding interface.

## Data Availability

The original contributions presented in this study are included in the article/supplementary material. Further inquiries can be directed to the corresponding author(s).
