# Supplementary material for: AscF in the mycobacterial CIII–CIV supercomplex lacks metal and nucleotide binding but links malate oxidation to respiration
Source: FEBS Lett. 2026 Jul 21;600(15):2129–42. doi: 10.1002/1873-3468.70414 (PMC13456599; doi:10.1002/1873-3468.70414)
Supplement: Supplementary file 1 [file FEB2-600-2129-s001.docx]

**Supporting Information**

**AscF in the mycobacterial CIII-CIV supercomplex lacks**

**metal and nucleotide binding but links malate oxidation to respiration**

Eni Rile^1#,^ Riccardo Diamanti^2^#, Daniel Lundin^2^, Soni Sharma^2^, Robin Lissner^1^, Coenraad P. Kuil^3^, Martin Högbom^2^*, Dirk Bald^1^*, Dan Sjöstrand^2^*

^1^Molecular Microbiology Section, A-LIFE and AIMMS, Faculty of Science, Vrije Universiteit Amsterdam, 1081 HZ Amsterdam, The Netherlands

^2^Department of Biochemistry and Biophysics, Stockholm University, 106 91 Stockholm, Sweden

^3^Medical Microbiology and Infection Control (MMI), Amsterdam University Medical Center Location VUmc, 1081 HZ Amsterdam, the Netherlands

#These authors contributed equally.

*Correspondence to:

Dan Sjöstrand (dan.sjostrand@dbb.su.se), Dirk Bald ([d.bald@vu.nl](mailto:d.bald@vu.nl)) or Martin Högbom ([martin.hogbom@dbb.su.se](mailto:martin.hogbom@dbb.su.se)).

**Figure S1: Expression constructs for protein purification.** (A) The *ascG* and *ascF* genes overlap in the M. smegmatis genome, and are thus likely expressed on the same polycistronic mRNA. (B) The construct for expression in E. coli is comprised of the codon optimised *ascG* and *ascF* genes, with a spacer sequence encompassing a ribosome binding site. (C) The AscG-∆NTMH protein, with residues 1-26 deleted.

**Figure S2: TPM domain hydrophobic core and metal binding comparison.** (A) Hydrophobic fold-preserving interactions are relatively well conserved between *P. gingivalis* 0361, *B. argentinensis* BA42 and *M. smegmatis* AscF (except for the second helix). (B) The N-terminal beta-strand of *A. thaliana* TLP18.3 is in AscF/AscG replaced by the AscG antiparallell beta-strand, and hydrophobic side chains occupy the space corresponding to the charged and polar side chains at the metal binding site. In *B. argentinensis* BA42, two Ca2+ ions are coordinated by residues Asn140, Asp136, Asp134 and Glu30. While the negatively charged Glu52 and Glu53 of AscF are similarly positioned to BA42 Asp136 and Asp134, this pattern is disrupted by a positively charged Arg65 in the approximate position of BA42 E30, rendering Ca2+ binding in this position unlikely.

**Figure S3: Structural alignments with other TPM domain proteins.**

Structural alignment (Software: DALI server) of *M. smegmatis* AscF with (A) *A. thaliana* atTLP18.3, (B) *P. gingivalis* pg0361, (C) *C. glutamicum* cg2496, (D) *B. argentinensis* BA42, (E) *B. argentinensis* BA41, (F) *R. marinus* Rhom172_1776 and (G) *M. tuberculosis* AscF. (Blue: *M. smegmatis* AscF template. Red: beta-strand portion of *M. smegmatis* AscG. Orange: the structurally equivalent beta-strand from the homologue). In A (partially also in E, F), but not in B, C, D the C-terminus is folded back over the core domain (solid arrow). In A the N-terminus also forms an additional helix (dashed arrow). In D, the N-terminus is truncated, and the beta-strand is instead formed by the C-terminus (red arrow). This is the same strand that in AscF is “replaced” by the antiparallell strand from AscG.

**Figure S4: Schematic representation of the ascF frameshift mutation.** A 7-bp deletion (ATGGACC) leads to a frameshift, resulting in a truncated AscF protein. Codons are shown in boxes with corresponding amino acid abbreviations below.

**Figure S5: RT-PCR analysis of *mqo* expression.** Transcript levels of *mqo* were normalized to *sigA* as reference gene, and are shown relative to wild-type. The data represent mean value ± SD from three independent experiments. Statistical analysis was performed by ordinary one-way ANOVA followed by Dunnett’s multiple comparisons test against the wild-type control. * p < 0.05; ns, not significant.

**Figure S6: Quantification of proton pumping activity from ACMA fluorescence traces.** Fluorescence intensity was normalized to an initial value of 100%. Maximum quenching was calculated as the difference between initial and minimum fluorescence intensity, expressed as a percentage of the initial value. Rate of quenching was determined by linear regression of the initial fluorescence decrease upon succinate addition. The data represent mean ± SD by ordinary one-way ANOVA followed by Dunnett’s multiple comparisons test against the wild-type control. **** p < 0.0001; ns, not significant.

**Figure S7: Analysis of the binding interface.** (A) Cryo-EM structure of the *M. smegmatis* AscF/AscG-Mqo complex, extracted from the Cryo-EM structure of the Mqo-bound *M. smegmatis* respiratory supercomplex. Green: Mqo, Pale blue: AscF, Red: AscG. (B) In silico prediction (Software: The Alphafold 3 server) of the complex, based on relevant parts of the individual proteins (see Materials and Methods for details). Green: Mqo, Pale blue: C-terminal portion of AscF (excluding the N-terminal extension), Red: C-terminal portion of AscG (excluding the TM helix. (C) The predicted binding interface, encompassing a putative 6-residue hydrophobic core and 6-residue charged zipper.
